# The GoldX Fiducial Eraser

**DOI:** 10.3390/ijms25137442

**Published:** 2024-07-06

**Authors:** Peter Van Blerkom, Armel Bezault, Cécile Sauvanet, Dorit Hanein, Niels Volkmann

**Affiliations:** 1 Department of Chemistry and Biochemistry, University of California Santa Barbara, Santa Barbara, CA 93106, USA; 2 Structural Image Analysis Unit, Department of Structural Biology and Chemistry, Institut Pasteur, Université Paris Cité, CNRS UMR 3528, 75724 Paris, France; 3 Institut de Biologie Intégrative de la Cellule, CNRS CEA, Université Paris Saclay, 91190 Gif-sur-Yvette, France; 4 Department of Biological Engineering, University of California Santa Barbara, Santa Barbara, CA 93106, USA; 5 Department of Electrical and Computer Engineering, University of California Santa Barbara, Santa Barbara, CA 93106, USA

**Keywords:** cryogenic electron tomography, fiducial marking, tomogram processing

## Abstract

Gold nanoparticles with sizes in the range of 5–15 nm are a standard method of providing fiducial markers to assist with alignment during reconstruction in cryogenic electron tomography. However, due to their high electron density and resulting contrast when compared to standard cellular or biological samples, they introduce artifacts such as streaking in the reconstructed tomograms. Here, we demonstrate a tool that automatically detects these nanoparticles and suppresses them by replacing them with a local background as a post-processing step, providing a cleaner tomogram without removing any sample relevant information or introducing new artifacts or edge effects from uniform density replacements.

## 1. Introduction

Electron tomography (ET) and cryogenic electron tomography (cryo-ET) are techniques whereby a series of images are taken in a transmission electron microscope (TEM) of the same region of interest at a variety of angles with the specimen rotated on a goniometer, also called a tilt series. In cryogenic conditions, this technique becomes incredibly powerful and is able to visualize the three-dimensional structure of vitrified hydrated biological samples in their native state with nanometer resolution. However, the reconstruction of the three-dimensional TEM densities in cryo-ET relies upon an accurate alignment of each image in the tilt series. For vitrified hydrated biological samples, the signal-to-noise ratio is very low, making inter-tilt alignment a challenge. To enhance alignment precision, small uniform electron-dense objects often in the form of gold nanoparticles in the size range of 5 to 15 nm can be introduced into the sample as tracking fiducials [1].

While tracking these fiducials is quite useful for alignment and is a standard method employed in tomographic reconstruction algorithms such as those provided in IMOD [2], their intense signal compared to the sample of interest introduces severe non-local artifacts such as streaking, corrupting the signal of nearby densities. These non-local artifacts are primarily caused by the fact that the tomographic reconstructions are derived from incomplete projection sets, usually covering only ±60 degrees, due to restrictions of the tilt geometry in the microscope. The resulting distortions are commonly referred to as missing-wedge artifacts. To alleviate the non-local effects of the fiducials, approaches were developed that can mask fiducials tracked by IMOD in the tilt images with a solid circle filled with the local grey level average [3], uniform random noise [4], or estimates derived from compressed sensing [5]. A more general approach that was developed for detecting and replacing contaminations caused by focused ion beam milling [6] can also be used to mask the fiducials but uses the overall grey level average of the tilt image. While these approaches do help in suppressing the fiducial artifacts, new non-local artifacts are introduced, which is caused by the missing wedge distortions of texture differences and sharp borders between the surrounding density and the replacement density. In addition, the approaches based on IMOD use a uniformly sized circle and the positions derived from tracking which can lead to incomplete masking if the size is chosen too small or inadvertently excluding regions containing vital structural details. Furthermore, fiducials that were not tracked will not be replaced.

The balance between the number of gold particles distributed within the area of interest and their usability for high-quality alignment is often quite delicate. The density of gold particles is determined empirically and can vary significantly between samples, grids, gold batches, and vitrification sessions. This complexity is heightened in cellular tomograms because of the potential obstruction of non-repeating unique features of interest by gold particles. Furthermore, the extensive hours invested in sample preparation and successful tomography sessions, compounded by the low frequency of cellular events identified in light microscopy for correlative light and electron microscopyy (CLEM), can lead to devastating results when gold particles obstruct the entities of interest.

To provide a method for resolving the issue of gold fiducial artifacts, we developed GoldX 0.1 , a program running within pyCoAn 0.3 [7], a python-based software package and interface designed specifically for interacting with electron microscopy data incorporating many of the standard datatypes and algorithms commonly used in data collection, reconstruction, and analysis built as an extension of the methods described for Correlative Analysis of electron microscopy data (CoAn) [8]. GoldX introduces a method that substitutes fiducial markers in a manner that solely replaces the marker and its defocus halo without extending masking beyond the markers’ distinct shape. Moreover, GoldX avoids generating non-local missing-wedge artifacts caused by texture differences or sharp borders of the replacement density, thereby facilitating analysis of areas within tomographic reconstructions that might otherwise be compromised by these artifacts.

## 2. Results

Tracking fiducials are commonly used to align tomographic tilt series because they offer better accuracy and ease compared to fiducial-free tracking protocols. However, the intense signals from gold fiducials can create artifacts such as streaking, which may obscure details from the regions of interest. To mitigate this, software like IMOD 4.11 provides methods to mask high-signal areas, but these can sometimes leave non-local artifacts, overlook defocus halos, or inadvertently mask crucial structural details. We developed GoldX within pyCoAn that delivers a different approach to address these challenges. This tool is designed to selectively remove fiducials and their direct effects, such as defocus halos, without affecting the surrounding areas, thus improving the analysis and data mining of tomographic reconstructions.

### 2.1. GoldX Workflow

**Figure 1:**
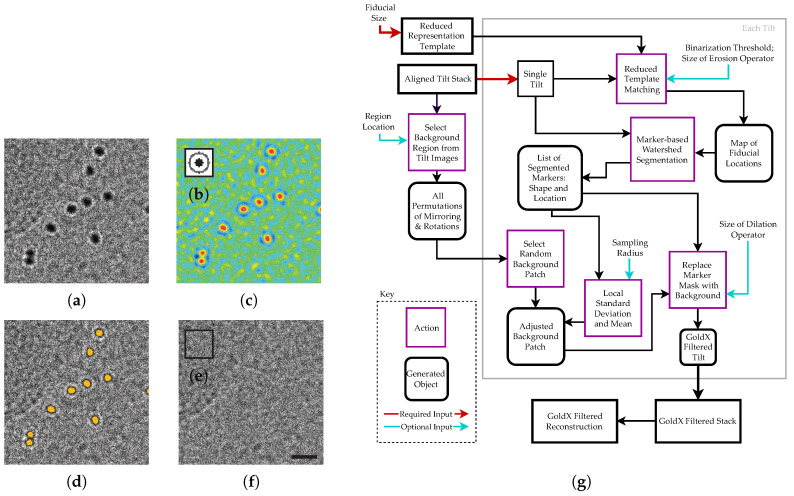
GoldX process for fiducial marker removal. A tilt section with gold fiducials (**a**) is analyzed using a reduced template matching method with template (**b**), where the peaks (**c**) are selected and filled using a watershed method (gold) (**d**) and masked using an adjusted background image (**e**), producing a fiducial-free image (**f**). A flow-chart of the GoldX procedure is shown in (**g**). Scale bar for (**a**–**f**) is 20 nm.

To process tomogram tilt series, the aligned tilt stacks are first imported to pyCoAn. From these imported stacks, substacks of the zero-degree tilt (Figure 1a) and maximal tilt are used for optional parameter optimization.

In the initial step of the GoldX processing workflow, the positions of the gold fiducials in the tilt images are determined. This process uses the reduced template concept [9] to produce a score map, which is then thresholded to provide a binary map. The binary map is post-processed using a morphological erosion operation with a circular structuring element to remove spurious peaks. Required input parameters are the pixel size and the size of the average size of the fiducials, which are necessary for adequate scaling of the software-provided reduced representation 2D template for fiducial detection (Figure 1b). The scaling factor can be fine-tuned manually such that the inner dark portion of the template optimally matches the size of the average particle and the outer light portion matches the defocus halo. The reduced template leaves a gap between the inner template points that detect the dark area and the thin outer ring of template points that detect the defocus halo. This design accounts efficiently for minor size and shape variations of the individual fiducial markers. The actual size of the fiducials does not affect performance, in our experience. The binary threshold and the size of the structuring element for the erosion operation are adjustable parameters even though the defaults work well for most cases. Adjustments tend to be most beneficial for thick samples where the contrast between the fiducials and the surrounding material is low.

For testing the quality of the fiducial detection with a particular set of parameters, location maps of the zero (Figure 1c) and maximal tilts were viewed in IMOD and compared visually to the original micrograph. The maximal tilts have the lowest contrast within the tilt series and good performance in the maximal tilt normally indicates good performance throughout the whole tilt series. The goal is to adjust the parameters to minimize both the false negative and false positive peaks in zero tilt as well as the maximum tilt. Generally, conditions can be found within two or three iterations that generate no false positives or false negatives in the zero tilt and either no or very few false hits in the maximum tilt image.

Next, the peak locations in the fiducial location map are used as markers for marker-based watershed segmentation to extract the masks for the fiducials. Briefly, the markers identify regions that are segmented from the original tilt-series images using the watershed algorithm [10]. This operation essentially segments a region corresponding to the exact shape and size for each individual fiducial (Figure 1d). This step is automatic and parameter-free. It should be noted that this step also takes care of aggregated fiducials as long as at least one of the border fiducials is detected. The watershed will then fill out the entire connected region using that marker.

To account for defocus halos, a morphological dilation operation with a circular structuring element is performed to expand the mask. The default parameter for the structuring-element size is set generously to ensure that the entire halo is included. Optionally, this parameter can be adjusted to provide a tighter mask if desired.

To test the masking quality, an initial background replacement section is generated with a constant value to provide a visible outline of where the desired GoldX-generated replacement would be substituted. This gives the necessary feedback to adjust the size of the structuring element used for the dilation operation to optimally match the fiducial and its associated defocus halo if desired.

Once the masks for the fiducials are determined, they can be replaced. The goal is to minimize any differences in noise statistics and texture and to avoid any recognizable borders between regions. Any difference propagates because of the missing wedge and potentially corrupts valuable information. Our approach is to select a featureless background square from the tilt series being processed. This ensures that texture and noise statistics are matched well. This square should be large in relation to the individual fiducials to avoid accidental correlation between replaced patches. Each patch replacement is picked randomly from this background. To further randomize, we pick randomly either from the original background or a transformation (mirroring, ±90° or 180° rotation, or a combination of rotation and mirroring). For automated operation, the program picks 50 regions randomly and finds the one with the lowest root-mean-square deviation. This region is usually sufficiently featureless and flat to serve as a suitable background. Alternatively, a background region can be selected manually. Before patch replacement, the picked background portion is scaled to match the mean and root-mean-square deviation of the region immediately surrounding the individual fiducial mask. A flow-chart of the entire process from fiducial picking to patch replacement is described in Figure 1g.

### 2.2. GoldX Processing Alleviates Fiducial Interference in Compromised Datasets

To showcase the effectiveness of GoldX, we selected two experimental tomography datasets: a reconstituted system of virus-like particles (VLPs) mixed with fiducials at the time of vitrification, and a cellular system where fiducials were incubated with Vero cells before vitrification. These two specific datasets were chosen due to their exceptionally high abundance of fiducials, which typically renders the tilt series unusable for further analysis. Because ground truth data were not available, we based our assessment on visual inspection.

**Figure 2:**
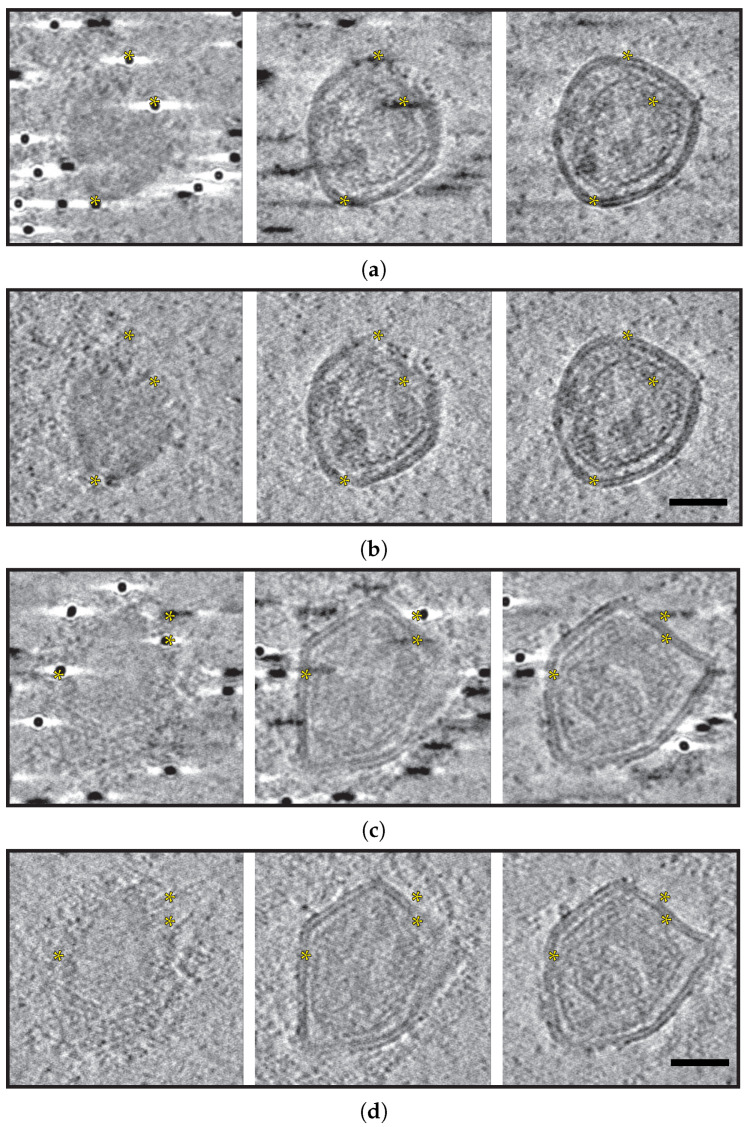
Gold removal of fiducials in a reconstituted system. Successive sections from selected tomograms of reconstituted systems imaged under cryogenic conditions are presented in (**a**–**d**). (**a**,**b**) display unprocessed sections, while (**c**,**d**) show sections processed with GoldX. Scale bars are 50 nm. Note that the gold particles and reconstruction artifacts that obscure possible regions of interest (yellow asterisks) in the unprocessed sections are displayed without compromising features following GoldX processing.

**Figure A1:**
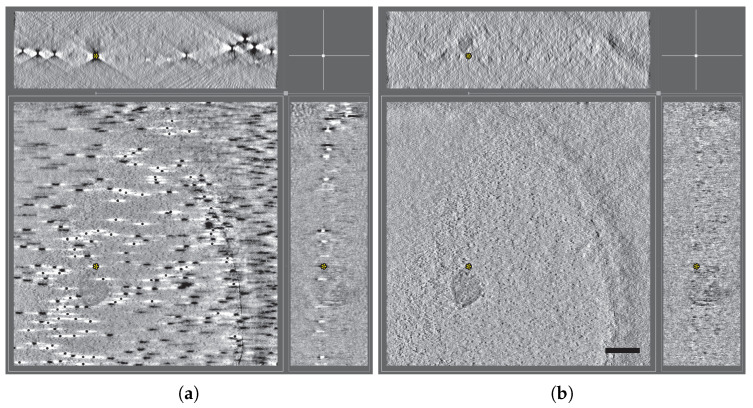
A tomogram section of a full micrograph, original tomogram (**a**), and GoldX-processed tomogram (**b**) with XZ and YZ sections (position at yellow asterisk) of reconstituted system. Scale bar is 100 nm.

For the reconstituted system, two examples of fiducials interfering particularly strongly with regions of interest were present in the reconstructed tomograms. Selected sections can be seen in Figure 2a,c. Note that dark streaks can be seen throughout even though these sections are removed in height from the physical location of the fiducial. XZ and YZ views in an uncropped section of Figure 2a,b can be seen in Appendix A Figure A1.

After GoldX processing, both reconstructed tomograms are free of such artifacts, even in areas where the fiducial was directly in contact with or adjacent to the VLP (Figure 2b,d). Videos traversing slice-by-slice through the 3D tomogram in these regions with and without GoldX processing can be found in Supplementary Videos S1 and S2.

**Figure 3:**
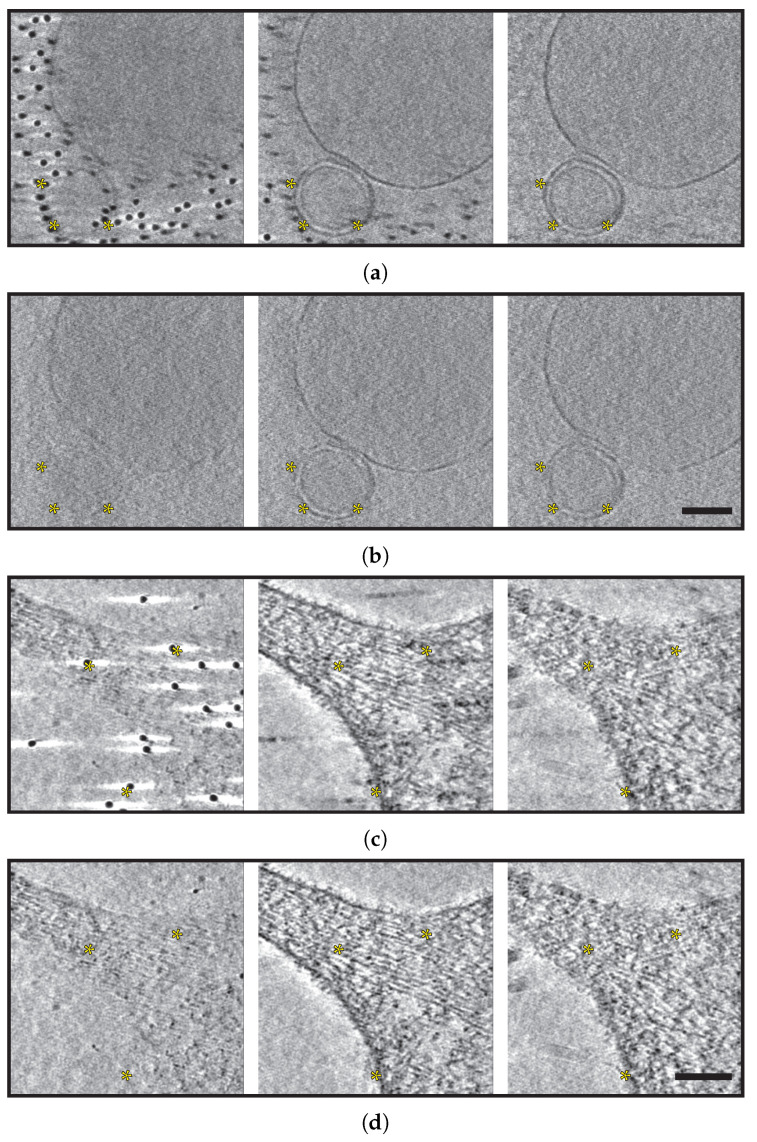
Gold removal of fiducials in cellular systems. Successive sections from selected tomograms of cellular systems imaged under cryogenic conditions are presented in (**a**–**d**). (**a**,**c**) display the unprocessed sections, while (**b**,**d**) show sections processed using GoldX. Each scale bar represents 50 nm for (**a**,**b**) and 100 nm for (**c**,**d**). Observe that in the unprocessed sections, gold particles and reconstruction artifacts obscure possible regions of interest (yellow asterisks). These same regions are displayed without any compromising features in the GoldX-processed sections.

For the cellular system, similar two examples of fiducials heavily interfering with regions of interest were selected. Two-dimensional slices of these regions can be seen in Figure 3a,c. Similar to the example of the reconstituted system, dark areas as artifacts from the fiducial densities can be observed and possibly be misinterpreted as protein densities, especially those artifacts close to membrane structures.

Once again, after GoldX processing, the reconstructed tomograms are free of such artifacts, and the visibility of different cellular structures both at the cellular edges and intracellular features are similarly improved (Figure 3b,d). Videos traversing slice-by-slice through the 3D tomogram in these regions with and without GoldX processing can be found in Supplementary Videos S3 and S4.

#### Comparison to Other Software

**Figure A2:**
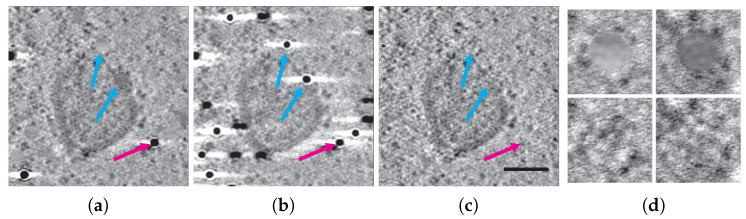
IMOD-processed tomogram section with tracked fiducials replaced with gray (**a**), original tomogram section (**b**), and GoldX-processed section (**c**). An example of a fiducial that was not tracked by IMOD and, therefore, not removed is indicated by the magenta arrow in (**a**). An example of tracked and removed fiducials with spherical outline visible in (**a**) is indicated by cyan arrows. Same regions are indicated in (**b**,**c**), artifacts are not present in (**c**). The scalebar for (**a**–**c**) is 50 nm. A 3× enlarged view of areas near the blue arrows is shown in (**d**). Upper panels are IMOD-processed, lower panels are GoldX-processed. Note that possible membrane extensions can be seen in the lower right corner while they are obscured in the upper right corner.

For comparison, the same aligned tilt series reconstructed as a tomogram in Figure 1a was processed using the IMOD 4.11 workflow to produce a fiducial masked tilt series, which was then reconstructed as the other datasets. The IMOD process requires the fiducials and tilt series to be constructed in the same IMOD workflow as opposed to the workflow-agnostic method of GoldX. Additionally, in IMOD, only the fiducials that can be tracked through the entire tilt series are masked (magenta arrow, Appendix A Figure A2a, fiducials indicated in unprocessed tomogram in Appendix A Figure A2b). IMOD also performs its masking by replacing the area with a uniform spherical region of either an average gray value or white noise and does not accommodate the variety of shapes and sizes the fiducials may adopt. Consequently, some artifacts remain in the IMOD-masked density even if the fiducial was tracked through the full tilt series (cyan arrow, Appendix A Figure A2a compared to GoldX, Figure A2c).

## 3. Discussion

Fiducial markers, although often seen as a necessity for high-quality alignment, are entities that can potentially take up a large amount of the high-signal real estate in cryo-ET tilt series. Even if sparse, they might end up near a region of interest, interfering with the interpretation and analysis of the reconstruction. Here, we demonstrated that GoldX is capable of removing these high-signal fiducials and the associated artifacts, providing clean tomograms with minimal loss of information even in samples where the fiducials are closely overlapping regions of interest. With their high contrast, fiducials are able to provide high alignment accuracy in low-contrast datasets such as those taken close to focus (and, therefore, containing higher resolution information). However, these are the exact situations where the artifacts from high-contrast objects will overwhelm the signal of the sample. With GoldX, fiducials can remain the gold standard of providing alignment features while allowing for high-resolution data collection.

An additional motivation for developing this approach aims at removing the artifacts caused by high-density markers in systems that contain gold-labeled antibodies to mark specific biomolecular complexes. The application of GoldX in such a scenario allows for maintaining the molecular densities of interest without distortions and artifacts caused by the directly attached gold particle. Similarly, if high-density labeling is used for single-particle analysis (SPA), GoldX can avert bias in 2D classification and alignment. GoldX may also allow for the use of a higher density of fiducials than commonly applied, allowing for a more accurate inter-tilt alignment while minimizing the risk of interference. The high density of fiducials may be useful for understanding and correcting for deformations occurring in samples over the acquisition of a tilt series.

## 4. Materials and Methods

### 4.1. Sample Preparation

#### 4.1.1. Vero Grids

Vero E6 cells (source ATCC CRL-1586) were seeded over holey carbon-film-coated gold EMBRA finder grids (R5/20 Au EMBRA Finder 200 mesh; N1-C43cAuE1-01; Quantifoil Micro Tools, Jena, Germany), or holey SiO_2_-film-coated gold grids (200 mesh R2/2; N1-S16nAu20-01; Quantifoil Micro Tools, Jena, Germany) following the procedures described in [11]. Photopatterning and protein functionalization of EM grids followed the procedure described in [12]. Following cell seeding (24 to 48 h course), cells were fixed using PHEM buffer as detailed in [13]. Upon completion of light microscopy survey, selected grids were slated for vitrifaction. Prior to vitrification, 0.5 to 1 μL of fiducial markers (10 nm gold nanoparticles (Cell Microscopy center, UMC Utrecht)) were added (~15 s incubation) to the samples. Vitrification was performed using either a Vitrobot or an EMGP (Vitrobot Mark IV; Thermo Fisher Scientific, Hillsboro, OR, USA; EMGP; Leica Microsystems, Wetzlar, Germany) using one side blotting (~15 s), plunge-frozen in liquid-nitrogen-cooled liquefied ethane, and stored in liquid nitrogen until investigation.

#### 4.1.2. VLP Grids

Virus-like particles (VLPs) were prepared by adapting the procedure described in [14]. Then, 5 μL of sample was then placed upon a holey carbon-film-coated copper grid (R1.2/1.3 400 Mesh; N1-C14nCu40-01; Quantifoil Micro Tools, Jena, Germany) pre-treated by plasma cleaning (Solarus II Plasma Cleaner; Gatan, Inc., Pleasanton, CA, USA). Prior to vitrification, 0.7 μL of fiducial markers (5 nm gold nanoparticles (Cell Microscopy center, UMC Utrecht)) was added to the samples (~15 s incubation). Vitrification was performed using a custom-build manual cryo-plunger and one side blotting (~30 s), manually plunge-frozen in liquid-nitrogen-cooled liquefied ethane, and stored in liquid nitrogen until investigation.

### 4.2. Electron Microscopy Data Collection

Vero grids were imaged on a Titan Krios G3i (Thermo Fisher Scientific, Hillsboro, OR, USA) operated at 300 keV equipped with K3 Summit direct electron detection device with a BioQuantum energy filter (Gatan, Inc., Pleasanton, CA, USA) operating in zero-loss mode with a slit width of 20 eV in electron counting mode (“vero sample 1”), or on a Glacios 1 (ThermoFisher, Hillsboro, OR, USA) operated at 200 keV equipped with Falcon 3 direct electron detector (ThermoFisher, Hillsboro, OR, USA) operating in linear mode (“vero sample 2”), while VLP grids were imaged on a Glacios using the electron counting mode. The linear mode was chosen for the first sample as part of a comparison between linear and counting modes. Tilt series were acquired using SerialEM 3.8.6 [15] (vero samples) or SerialEM 3.6 (VLP samples). Each tilt was collected at a pixel size of 4.59 Å/px (vero sample 1), 4.05 Å/px (vero sample 2), or 1.98 Å/px (VLP samples). Tilts were collected from 60° to −60° using a dose symmetric tilt scheme every 3°, totaling 41 tilts [16]. Each tilt was collected as a series of 13 frames (vero samples) or 14 frames (VLP samples), with a total accumulated dose for all samples at ~100 e^−^/Å^2^.

### 4.3. Data Processing

#### 4.3.1. Tomographic Reconstruction Procedure

The original tilt series were aligned using the patch-based method implemented in Aretomo [17] with 4 × 5 overlapping patches. For the GoldX-processed tilt series, the alignment parameters of the respective original tilt series were used. Reconstructions were calculated using the simultaneous iterative reconstruction technique implemented in tomo3D [18]. The resulting reconstructions were enhanced in pyCoAn using a Wiener-like filter accounting for the contrast transfer function and an estimate of the spectral signal-to-noise ratio [19].

#### 4.3.2. GoldX Procedure

GoldX processing and parameter optimization were performed in the interactive python shell provided with the pyCoAn package. GoldX provides sensible defaults for all steps of the procedure, allowing for fully automated processing for well-behaved tilt series. GoldX also allows for optional fine-tuning of the various steps to optimize performance. An The evaluation of the individual processing steps is facilitated by the IMOD 3dmod GUI, with template scaling validated in UCSF Chimera [20]. After reconstruction, the 3dmod GUI can be used to compare the unprocessed and GoldX-processed reconstructions to evaluate for artifacts and retention of detail. Generally, the default parameters perform well, and no more than two or three iterations of this procedure are required to obtain the best possible performance in cases where optimization is required.

## Data Availability

Data are contained within the article and supplementary materials.

## Supplementary Materials

The following supporting information can be downloaded at: https://www.mdpi.com/article/10.3390/ijms25137442/s1.

## Abbreviations

The following abbreviations are used in this manuscript:

ET	Electron tomography
cryo-ET	Cryogenic electron tomography
TEM	Transmission electron microscope
CLEM	Correlative light and electron microscopy
VLP	Virus like particle
SPA	Single-particle analysis
2D	Two-dimensional
